# Natural Products for the Immunotherapy of Glioma

**DOI:** 10.3390/nu15122795

**Published:** 2023-06-19

**Authors:** Qi Huang, Xier Pan, Wenhao Zhu, Wen Zhao, Hongzhi Xu, Kaili Hu

**Affiliations:** 1 School of Pharmacy, Shanghai University of Traditional Chinese Medicine, Shanghai 201203, China; qi_huang2023@163.com (Q.H.); panxier2022@163.com (X.P.); 2 Institute of Interdisciplinary Integrative Medicine Research, Shanghai University of Traditional Chinese Medicine, Shanghai 201203, China; 3 Department of Anaesthesiology, Huashan Hospital, Fudan University, Shanghai 200040, China; zhuwenhao91@163.com (W.Z.); 18501649871@163.com (W.Z.); 4 Department of Neurosurgery, Huashan Hospital, Shanghai Medical College, Fudan University, Shanghai 200040, China; 5 National Center for Neurological Disorders, Shanghai 200040, China; 6 Shanghai Key Laboratory of Brain Function and Restoration and Neural Regeneration, Shanghai 200040, China; 7 Neurosurgical Institute, Fudan University, Shanghai 200040, China; 8 Shanghai Clinical Medical Center of Neurosurgery, Shanghai 200040, China

**Keywords:** natural products, immunotherapy, glioma, tumor immunosuppressive microenvironment

## Abstract

Glioma immunotherapy has attracted increasing attention since the immune system plays a vital role in suppressing tumor growth. Immunotherapy strategies are already being tested in clinical trials, such as immune checkpoint inhibitors (ICIs), vaccines, chimeric antigen receptor T-cell (CAR-T cell) therapy, and virus therapy. However, the clinical application of these immunotherapies is limited due to their tremendous side effects and slight efficacy caused by glioma heterogeneity, antigen escape, and the presence of glioma immunosuppressive microenvironment (GIME). Natural products have emerged as a promising and safe strategy for glioma therapy since most of them possess excellent antitumor effects and immunoregulatory properties by reversing GIME. This review summarizes the status of current immunotherapy strategies for glioma, including their obstacles. Then we discuss the recent advancement of natural products for glioma immunotherapy. Additionally, perspectives on the challenges and opportunities of natural compounds for modulating the glioma microenvironment are also illustrated.

## 1. Introduction

The current clinical treatment methods for glioma include surgical resection, radiotherapy and chemotherapy. Although the tumor is treated with a combination of various methods, the disease progresses rapidly, with an average recurrence of 8–9 months after diagnosis, and an average survival period of only 15 months [1]. Cancer immunotherapy uses the body’s immune system to achieve the purpose of preventing, controlling, and eliminating cancer [2]. The key to glioma immunotherapy is reversing the tumor immunosuppressive microenvironment, and eliciting effective immune responses. Gliomas are called immunologically “cold” tumors [3]. In the past decades, it has been believed that they have an immune escape phenomenon to normal immune responses and can avoid the surveillance of immune cells, including microglia, T cells, natural killer cells (NK cells), and macrophages. Although antigen pathways from the brain to the deep cervical lymph nodes were first identified in the 1980s [4,5,6,7], a direct drainage route for cerebrospinal fluid containing immune cells from the cervical lymph nodes was not discovered until 2015 with the discovery of functional lymphatic vessels in the meninges [8,9,10], which has provided the basis for immunotherapy of glioma.

Central nervous systems have always been considered immune privileged historically, mainly due to the lack of a traditional lymphatic system, which will only produce a very weak immune response [11]. However, with the abolition of the concept of “immunity privilege” in the central nervous system and the proof of the lymphatic system in the brain, the immunology of the central nervous system has begun to rise [9,10,12]. Several immune cells in the central nervous system, including microglia and monocyte-derived macrophages, play a major role in the immune response [13]. However, in glioma, the brain’s immune system is in a suppressed state. The microglia, which are resident immune cells in the brain, will not kill tumor cells, they become tumor-associated microglia/macrophages (TAMs) in gliomas and typically exhibit an anti-inflammatory M2-type, and release cytokines, such as epidermal growth factor (EGF) and vascular endothelial growth factor (VEGF) to promote tumor growth [14]. In addition, C-C motif chemokine 2 (CCL2), colony-stimulating factor 1 (CSF-1), CX3CL1, and EGF secreted by glioma cells can promote the recruitment of TAMs and promote the transformation of the M2-type and lead to an immunosuppressive tumor microenvironment, further promoting the growth of glioma cells. At the same time, glioma cells secrete indoleamine-2, 3-dioxygenase (IDO) to deplete tryptophan to inhibit T cell activation [15]. M2-type macrophages secreted interleukin-6 (IL-6), interleukin-10 (IL-10), and TGF-β to promote the recruitment of regulatory T cells (Tregs) and inhibit the activation of effector T cells. Of course, the M1-type of TAMs secrete interleukin-1β (IL-1β) and tumor necrosis factor (TNF-α) to promote T cell activation to kill glioma cells, but this effect is minimal in the glioma microenvironment [16]. Transforming growth factor beta (TGF-β) secreted by glioma cells inhibits the activation of NK cells. Astrocytes in the glioma microenvironment also secrete cytokines such as TGF-β and IL-10 to inhibit T cell activation [16,17,18,19,20]. Therefore, reversing the immunosuppressive state is particularly important for the treatment of gliomas. Tumor immunotherapy mainly regulates the body’s immune response to achieve tumor killing. The current immunotherapy strategies for glioma mainly include vaccine therapy, chimeric antigen receptor T-cell (CAR-T cell), immune checkpoint inhibitors (ICIs), and virus therapy. However, glioma immunosuppressive microenvironments together with glioma heterogeneity and antigen escape limit the effectiveness of these immunotherapies.

Natural products have the characteristics of diverse biological activities, but are less toxic [21]. They have an important contribution to the treatment of glioma [22]. Moreover, many natural products have good immunomodulatory effects [23]. There are several ways by which natural products regulate the immune system and remodel the glioma microenvironment for glioma immunotherapy. This review describes the current approaches and major obstacles in glioma immunotherapy, with emphasis on the role of natural products in glioma immunotherapy, aiming to provide a better approach to glioma therapy.

## 2. Current Status of Immunotherapy for Glioma

Current clinical trials of glioma immunotherapy which include ICIs, vaccines, CAR-T cells, and virus studies result in many promising clinical results. It is noteworthy that ICIs can significantly increase the infiltration of immune cells and significantly prolong survival, which make it a promising therapeutic approach in glioma immunotherapy. However, the immunosuppressive microenvironment is still an important reason for the failure of glioma immunotherapy.

### 2.1. ICIs

Immune checkpoints are an important obstacle to the immunotherapy of gliomas. An immune checkpoint signaling pathway (PD1/PDL1) was responsible for inactivating and even killing activated T cells. Therefore, by blocking these signaling molecules, T cells can be continuously activated [24]. In recent years, the emergence of ICIs has brought a turning point to the immunotherapy of glioma, which brings hope to glioma patients. A clinical study of neoadjuvant PD1 blocking antibody pembrolizumab therapy in the treatment of recurrent glioblastoma showed neoadjuvant pembrolizumab, with continued adjuvant therapy following surgery, has significantly improved overall survival and progression-free survival compared with randomized to receive adjuvant, post-surgical PD-1 blockade alone; the median overall survival of patients in the new adjuvant group was 189 days longer than that in the pembrolizumab alone group, and the median progression-free survival was 27 days longer. Neoadjuvant pembrolizumab therapy induces the functional activation of tumor-infiltrating lymphocytes, and interferon-γ infiltration was also found in the glioma microenvironment [25]. In a single-arm phase II clinical trial (NCT02550249), Schalper et al. used the neoadjuvant drug nivolumab to prove that the nivolumab can increase the expression of chemokine transcription and increase immune cell infiltration, thereby supporting the local immunomodulatory effect [26]. ICI as a new glioma treatment method has attracted great attention. However, although it is one of the most promising approaches for tumor immunotherapy, the “cold phenotype” of gliomas makes the clinical application of ICI challenging. Intrinsic tumor resistance, such as the WNT-β-catenin signaling pathway, prevents antitumor immune responses by promoting the release of the immunosuppressive cytokine IL-10 and is a major challenge in ICI therapy for glioma [27,28]. In addition, the possibility of systemic side effects from immune checkpoint blocking therapy, such as rash, colitis, hypophysitis, hepatitis, pancreatitis, iridocyclitis, lymph node enlargement, neuropathy and nephritis, etc., remains an issue to be considered [29].

### 2.2. Vaccine

In recent years, vaccines have been a hot spot in immunotherapy and a good supplement to glioma treatment strategies. Vaccine immunotherapy is based on the personalized characteristics of tumors to induce tumor cell-specific immune responses [30]. It can prolong the survival of patients and improve the quality of life of patients. Glioma tumor vaccines mainly include peptide vaccines and dendritic cells vaccines, which induce immune responses by increasing the recruitment of antigen-specific T cells [31]. Wen et al. demonstrated that in a phase II clinical trial of newly diagnosed glioblastoma patients using the dendritic cells vaccine ICT-107 showed a 2.2 month increased progression-free survival (PFS) while maintaining the quality of life. In this trial, IFN γ immune response test showed an increase in responders treated with ICT-107 (50%) compared with control (33%) [32]. In addition, in a phase I/II clinical trial of peptide vaccine IMA950/poly-ICLC for the treatment of malignant astrocytoma, Migliorini et al. found that 63.2% of patients showed tumor peptide-specific CD8^+^ T cell response, and 36.8% of patients showed polypeptide CD8^+^ T cell response [33]. The advent of cancer vaccines represents a major opportunity for the treatment of gliomas. However, glioma heterogeneity is a major challenge for vaccine therapy. Gliomas demonstrate a natural tendency of harboring multiple tumor cell populations which have a unique set of mutations, and it is highly unlikely that targeting a single antigen will result in successful tumor control [34].

### 2.3. CAR-T Cell Therapy

Chimeric antigen receptor (CAR) technology is one of the most promising approaches in T cell therapy. It received the first batch of approval from the U.S. Food and Drug Administration (FDA) in 2017, completely changing the treatment and management of hematological malignancies [35]. Hegde et al. constructed a bispecific CAR molecule (TanCAR) that can specifically recognize glioma-related antigens HER2 and IL-13Rα2. TanCAR T cells can reduce antigen escape, enhance antitumor effects, and improve the survival rate of orthotopic transplantation of glioblastoma in mice [36]. A study showed that CAR-T cells in combination with IL-12 therapy not only enhanced the cytotoxicity of CAR-T cells, but also remodeled TMEs, promoted pro-inflammatory CD4^+^ T cell infiltration, and reduced Treg cell numbers [37]. CAR-T cell therapy is a breakthrough in the treatment of glioma, which can significantly enhance the cytotoxicity of T cells. However, a clinical trial demonstrated that EGFRvIII expression was deficient in tumor tissue after treatment with EGFRvIII CAR-T cells in more than 50% of patients [38]. Antigenic escape of gliomas can lead to tumor recurrence after CAR-T cell-directed therapy with tumor antigen, and is a major challenge for CAR-T cell therapy [36]. Moreover, the proliferation of over-activated CAR-T cells, which may cause cytokine storm syndrome leading to death, is an important obstacle to CAR-T cell development [39].

### 2.4. Virus Therapy

Oncolytic virus therapy uses viruses to infect and destroy tumor cells. The infected cancer cells are destroyed, while new infectious virus particles are released and further destroy the remaining tumor cells. Virus therapy then activates the immune system, which in turn encourages the immune cells to attack the tumor. Thus, oncolytic virus therapy can increase immune cell infiltration and induce inflammation within the TME, which is critical for breaking immune tolerance. The FDA approved Talimogene laherparepvec (T-VEC) in 2015 for treating metastatic melanoma with genetically modified herpes simplex viruses (HSVs) [40]. A study on the treatment of glioblastoma with Zika virus (ZIKV), an oncolytic virus, showed that ZIKV virus can target glioblastoma stem cells in vitro and has little effect on normal nerve cells. At the same time, in vivo experiments indicate that when tumors are inoculated with the ZIKV strain, it inhibits tumor growth in tumor-bearing mice and prolongs their survival, the median survival improved by about 10 days [41]. Another phase I study of DNX-2401 oncolytic adenovirus therapy for recurrent gliomas found that 20% of patients survived more than 3 years after a single intratumoral injection of DNX-2401, and the tumor volume of three patients was reduced by more than 95%. The tumor regression induced by DNX-2401 is caused by the direct oncolytic effect of virus infection [42]. Angelova et al. in a phase I/IIa study demonstrated that activated cytotoxic T lymphocytes and TAMs can be recruited into malignant glioma tumors when oncolytic parvovirus is administered systemically [43]. Although some oncolytic viruses have shown the ability to infect glioma, systemic use of oncolytic viruses remains limited. Oncolytic virus therapies must be optimized for patients with immunosuppressive TME and high glioma heterogeneity. In addition, some oncolytic viruses may also replicate in normal cells and cause damage. For example, T-VEC may lead to potential infections and cause long-term neurological and immune adverse events [44], such as fever and cellulitis. Therefore, the safety of viral therapy remains controversial.

### 2.5. Major Obstacles in Immunotherapy of Glioma

**Figure 1:**
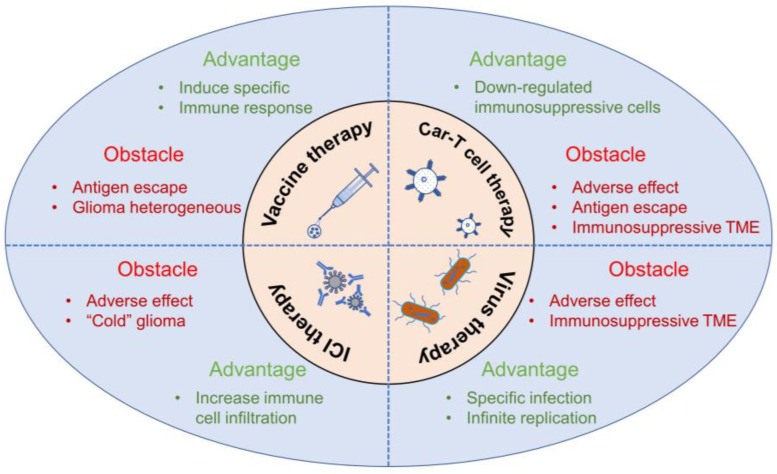
Advantages and obstacles of the current glioma immunotherapy. Current immunotherapy in glioma consists of immune checkpoint inhibitors (ICIs), vaccines, CAR-T cell therapy, and virus therapy. Although these strategies are beneficial to some glioma patients, there are still major obstacles thwarting the clinical application of glioma immunotherapy, including the heterogeneous, immunosuppressive TME, antigen escape, and adverse effects.

Although the advent of immunotherapy has brought hope to the treatment of gliomas, there are still great challenges for current glioma immunotherapeutics. The heterogeneity of gliomas, antigen escape, and immunosuppressive microenvironment are major obstacles to glioma immunotherapy, as described in Figure 1.

#### 2.5.1. Glioma Heterogeneity

Glioma is a heterogeneous tumor with different proliferative potential, invasive, histological grade, and clinical behavior [45], and glioma heterogeneity has been defined as an important cause of drug resistance, recurrence, and an important obstacle encountered in immunotherapy. Sampson et al. demonstrated that 82% of patients treated with the EGFRvIII peptide vaccine showed EGFRvIII expression loss when the tumor recurred [46]. Future directions in vaccine therapy may require targeting multiple epitopes to counteract the inherent heterogeneity of gliomas. In addition, a clinical study by biopsy with multiple regions of the tumor from a patient treated with EGFRvIII CAR-T cells demonstrated that the degree of EGFRvIII expression varied widely in different regions of the tumor, indicating that there are different degrees of the efficacy of CAR-T cells at different tumor locations [47]. The tumor heterogeneity of glioma leads to the development of immunotherapy resistance and reduces the efficacy of immunotherapy [48]. Therefore, for glioma immunotherapy, targeting tumor heterogeneity will be the key point. The appropriate treatment can be selected based on the specificity of each tumor or synergistic therapy can be attempted.

#### 2.5.2. Glioma Antigen Escape

Antigen escape includes antigen loss or downregulation. The tendency of glioblastoma to adapt rapidly through antigen escape remains a major obstacle to vaccine therapy and CAR-T cell therapy. Vaccine therapy can be affected by antigen escape, resulting in low immunogenicity of the vaccine and unsatisfactory antitumor immune response [49]. CAR-T cell therapy faces several challenges, including the possibility of tumor resistance to single antigen-targeted constructs [50]. Even though a single antigen targeting CAR-T cells may initially provide a high response rate, malignant cells in many patients show partial responses or complete loss of target antigen expression after treatment. The decrease in the antigen density of tumor cells is sufficient to evade the treatment of CAR-T cells. However, CAR-T cells require a minimum antigen density threshold to achieve therapeutic efficacy. Therefore, overcoming antigen escape is a promising strategy in glioma immunotherapy.

#### 2.5.3. Glioma Immunosuppressive Microenvironment (GIME)

During the oncogenesis process, the tumor microenvironment is heavily influenced by the inflammatory response. Immune cells play an important role in glioma proliferation and migration. In this microenvironment, apart from glioma cells, there exist two kinds of immune cells, including immunosuppressive cells, e.g., myeloid-derived suppressor cells (MDSCs), M2-type TAMs, and Tregs, and immune effector cells e.g., T cells and NK cells. M2-type TAMs, MDSCs, and Tregs maintain an immunosuppressed state and promote glioma growth, invasion and metastasis. T cells and NK cells are the predominant effector cells in innate and adaptive immunity, respectively. Tumor immunosuppressive microenvironments suppress the activity of T cells and NK cells, which makes it difficult for the immune system to attack and destroy tumor cells. This has become an important obstacle in glioma immunotherapy. So, how to activate immune cells and inspire the immune response is tremendously significant for the treatment of glioma. Furthermore, in the course of immunotherapy, many obstacles can arise in the GIME, including immunosuppressive cytokines and immunosuppressive immune cells [51]. Cytokine networks in the glioma microenvironment include prostaglandins E2, IL-6, IL-10, and TGF-β, each of which inhibits T cell proliferation and activation [52]. These immunosuppressive factors are consistently upregulated in surgical specimens of patients treated after immunotherapy compared to untreated glioma which further worsens the situation [48].

## 3. Natural Products for Immunotherapy of Glioma

**Figure 2:**
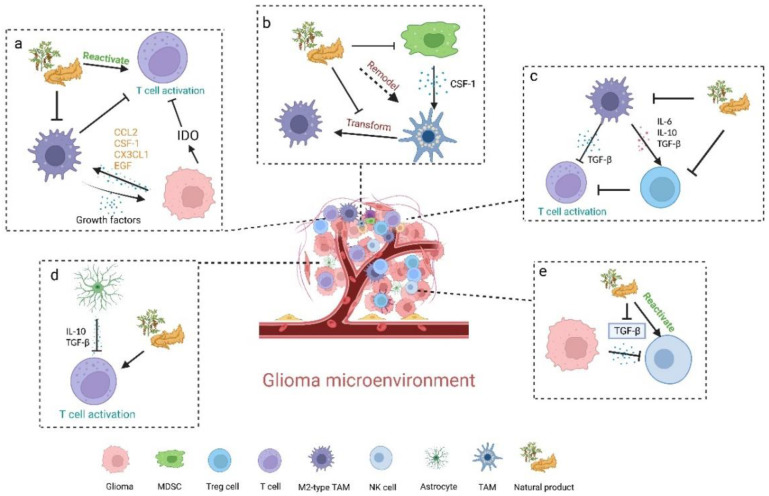
Natural products remodeling the immunosuppressive glioma microenvironment. (**a**) Glioma cells secrete CCL2, CSF-1CX3CL1, and EGF to promote M2-type TAM to inhibit T cell activation, M2-type TAM secretes growth factors to promote the growth of glioma cells, meanwhile, glioma cells can induce IDO to inhibit T cell activation. Natural products can inhibit M2-type TAM and reactivate T cells. (**b**) MDSC secretes CSF-1 to promote the transformation of TAM into M2-type. Natural products can inhibit the effect of MDSC on TAM and remodel TAM and inhibit the transformation of TAM to M2-type TAM. (**c**) M2-type TAM secretes TGF-β to inhibit T cell activation and secretes IL-6, IL-10, and TGF-β to promote the number of Treg cells and then inhibit T cell activation. Natural products can inhibit M2-type TAM and reduce the number of Treg cells. (**d**) Astrocytes secrete IL-10 and TGF-β to inhibit T cell activation, while natural products promote T cell activation. (**e**) Glioma cells secrete TGF-β to inhibit NK cell activation, and natural products can inhibit the secretion of TGF-β by glioma cells and reactivate NK cells. Created with BioRender.com (accessed on 17 May 2023).

Currently, since modern immunotherapy for the treatment of gliomas remains an unmet need, researchers have turned to natural products for better therapeutic efficiency and drug safety. Compared with chemotherapeutics, natural products display pharmacological effects based on the action mode of multicomponent-multichannel-multitarget, and more importantly, they have fewer side effects [22]. Natural products can regulate the immune system, playing an excellent role in the immunotherapy of glioma through the following mechanisms: (i) remodeling TAMs, (ii) inhibiting MDSCs and Tregs, (iii) reactivating immune effector cells (including T cells and NK cells), and (iv) modulating immune-related signaling pathway in glioma cells (Table 1 and Figure 2).

### 3.1. Natural Products Remodeling TAMs

It is widely acknowledged that TAMs can polarize into different phenotypes, including antitumor M1-type and pro-tumor M2-type, depending on various cytokines in the glioma microenvironment. Interferon-γ (IFN-γ) or lipopolysaccharide (LPS) can induce M1-type macrophage activation, which comes with the enhancement of inducible nitric oxide synthase (iNOS), IL-6, IL-12 levels, and Th1 immune response [53,54]. Conversely, IL-4 and IL-13 can bind to the receptor subunit IL-4Rα for M2-type macrophage activation, which is characterized by augmented expression of arginase 1, mannose receptor and IL-10. In GIME, TAMs tend to be M2 phenotype and secrete various immuno-suppressive factors to promote tumor growth and metastasis, thus exhibiting their potential to become a target in glioma immunotherapy.

#### 3.1.1. Chlorogenic Acid

Chlorogenic acid (3-O-caffeoylquinic acid, CHA) is a phenolic compound with a small molecular weight, widely distributed in many plants, such as honeysuckle, eucommia and hawthorn. Previous studies have reported that CHA has various beneficial pharmacological activities, such as antibacterial, anti-inflammatory, antioxidant and antitumor effects [55]. Among them, its antitumor and immune-regulation function attract increasing attention in cancer immunotherapy, especially for glioma [56,57].

Xue et al. explored whether the CHA-mediated antiglioma effects correlated with TAM polarization in GBM progression [58]. In vitro experiments showed that CHA treatment upregulated M1 markers (iNOS, MHC II and CD11c) induced by LPS/IFN-γ and downregulated M2 markers (arginase and CD206) induced by IL-4 in TAMs. The results suggested that CHA reprogrammed macrophage polarization from M2 to M1 phenotype by promoting STAT1 activation and inhibiting STAT6 activation, eventually inhibiting the growth of tumor cells. The activation of STAT1 and STAT6 pathways plays a significant role in TAM polarization. The in vivo experiment results presented that CHA treatment increased the proportion of CD11c-positive M1 TAMs in G422 xenograft mice and significantly reduced tumor volume [58]. However, the immune therapeutic effects of CHA were limited to rapid clearance in vivo and low accumulation in tumors [59]. Thus, Ye et al. employed mannosylated liposomes to fulfill a safe and targeted delivery of CHA for the treatment of GBM [60]. The liposomes had preferential accumulation in tumors and directed CHA to M2-type TAMs via the binding of mannose and mannose receptors overexpressed on M2-type macrophages. It was proven that the CHA-encapsulated liposomes had the potential to induce TAM polarization to the M1 phenotype and improve the immunotherapeutic effects on GBM.

Additionally, since CHA has a short half-life in blood circulation, standard injections are typically administered intramuscularly once a day and the clinical treatment needs for months or even longer in the clinic, leading to poor compliance of GBM patients [56,59]. Given that, one study fabricated CHA-phospholipid complex (CHA-PC)-loaded PEGylated liposomes (CPPL) for GBM treatment to reduce the administration frequency [56]. The CPPL exhibited an enhanced accumulation in the tumor site and inhibited tumor growth when the administration interval was prolonged to 4 days. However, the free CHA solution or CHA-PC-encapsulated liposomes showed antitumor effects only with once-daily administration. In addition, they also found that the antiglioma effects may be attributed to its ability to reverse the immunosuppressive microenvironment by suppressing MDSCs, downregulating Th2-related factors, and enhancing T-cell number. Surprisingly, both free CHA solution and CHA-PC-encapsulated liposome markedly facilitated the expression of MHC II, which is closely associated with the antitumor effects of TAMs, while CPPL failed to change the expression of MHC II, indicating that PEGylated preparation induced a different immune response [56].

#### 3.1.2. Curcumin

Curcumin (CC) is a polyphenolic component extracted from the rhizome of *Curcuma longa* L. and receives public attention due to its excellent pharmacological activity in the treatment of neurodegenerative diseases, inflammatory disorders as well as a wide spectrum of tumors including glioma [61,62].

Unfortunately, notwithstanding the marked antitumor efficacy of CC in vitro, the in vivo antitumor effects of CC are severely restricted by poor bioavailability, attributed to its physicochemical properties (such as hydrophobicity and short half-life) [63]. Therefore, Mukherjee et al. simultaneously prepared CC-CD68Ab (a glioblastoma-targeted adduct of CC) and Curcumin Phytosome (a lipid-encapsulated formulation of CC) to investigate the effect of CC on tumor remission and polarization of TAMs [64]. The results showed that both forms of CC exhibited the ability to induce TAMs to become M1 phenotype (Arginase1^low^, iNOS^high^) and suppress M2 phenotype (Arginase1^high^, iNOS^low^). In addition, they observed a marked increase in activated NF-κB (P-Ser276-p65) in the CC-treated GBM-associated Iba11 microglia. It suggested that CCs suppressed the nuclear localization of the NF-κB p50-homodimer that promoted TAMs to M2-type, and facilitated activation of the p50/p65 NF-κB that was linked to macrophage polarization to M1-type [64].

Moreover, one study designed a synergistic strategy (namely TriCurin) to achieve superior antitumor potency by combining CC with two other natural polyphenols, which are epicatechin gallate from green tea and resveratrol from red grapes and then fabricated liposome-encapsulated TriCurin (TrLp) for reversing GIME and enhancing GBM therapy [65]. The in vitro results show that TrLp could induce TAMs to tumoricidal M1 phenotype and facilitate the recruitment of NK cells. The intratumor presence of antitumor immune cells correlated with the apoptosis of GBM and GBM stem cells, indicating TrLp had the potential to become an immunotherapeutic agent against GBM [65].

#### 3.1.3. Apigenin

Apigenin is a flavonoid extracted from the Brazilian plant *Croton betulaster Müll*., which displays antiglioma effects by inhibiting proliferation, inducing differentiation and changing the inflammatory characteristics of glioma cells. The immunoregulatory effects of apigenin are exerted by modulating the immune profiles of cytokines, such as IL-10 and TNF [66].

Coelho1 et al. confirmed that apigenin could recover the immune properties of microglia against glioma cells via various pathways and targets [67]. They reported the reduced expression of CD206 (M2 profile marker) on microglia by apigenin treatment, and the increased expression of OX-42 and iNOS (M1 phenotypic markers), indicating the role of apigenin in increasing microglia-activated phenotype. In addition, C6 glioma cells had a low tumor migration and viability due to the marked reduction in IL-6 levels when incubated with a conditioned medium of microglia treated with apigenin. Furthermore, they found that microglial cells treated with apigenin are activated and endowed with chemotaxis toward the glioma. They also found that apigenin decreased glioma cell viability and promoted microglia differentiation, which correlated with the balance of microglia-derived TNF and IL-10 [67].

#### 3.1.4. Ginsenoside Rg3

Ginsenoside Rg3 is one of the main antitumor components in *Panax ginseng* C. A. Meyer. It shows excellent efficacy in inhibiting tumor infiltration, proliferation and metastasis [68,69]. Ginsenoside Rg3 was demonstrated to show robust immunoregulatory effects on reversing the GIME. Zhu et al. fabricated a liposome system co-loading ginsenoside Rg3 and anticancer drug paclitaxel (Rg3-PTX-LPs) to achieve synergistic antiglioma effects [70]. They found that Rg3-PTX-LPs exhibited a stronger antitumor efficacy than paclitaxel-loaded cholesterol liposomes and could prolong the survival time of intracranial C6 cell transplantation mice by reactivating the immunosuppressive microenvironment in glioma. TAM repolarization is a significant therapeutic mechanism of ginsenoside Rg3-based liposomes. The in vivo results showed the administration of Rg3-PTX-LPs decreased the M2-type population (CD206^+^) while increasing the M1 population (iNOS^+^), suggesting that Rg3-PTX-LPs exhibited an excellent ability to re-educate TAMs from pro-tumor M2 to antitumor M1. Apart from TAM repolarization, Rg3-PTX-LPs also inhibited Tregs and MDSCs, and promoted the expansion of CD8^+^ T cells to reverse the GIME [70].

#### 3.1.5. Rutin and Its Aglycone Quercetin

Widely found in various vegetables, fruits, and other plants, the flavonoids rutin and its aglycone quercetin are demonstrated to be able to reduce the viability of highly proliferative human glioblastoma multiform cells [71,72]. A previous report showed that rutin and quercetin inhibited the proliferation and migration of rat C6 glioma cells [73]. The natural compounds also induced the microglial chemotaxis that related to (i) upregulating TNF and downregulating IL-10 at protein and mRNA expressing levels, (ii) increasing mRNA expression of chemokines, such as CCL2, CCL5 and CXCL1, and (iii) promoting the mRNA expression of growth factors, such as heparin-binding growth factor (HBGF), insulin-like growth factor (IGF) and glial cell-derived neurotrophic factor (GDNF). Rutin and quercetin also directed microglia toward an inflammatory profile with upregulated expression of mRNA for IL-1β, IL-6 and IL-18, and reduced expression of mRNA for arginase, TGF-β, nitric oxide synthase 2 (NOS2) and prostaglandin-endoperoxide synthase 2 (PTGS2). These results indicated that antiglioma effects of rutin and quercetin correlated with the modulation of microglia inflammatory profile and these flavonoids could be considered for preclinical and clinical studies as adjuvant molecules for immunosuppressive microenvironment modulation of glioma [73].

### 3.2. Natural Products Inhibiting MDSCs and Tregs

MDSCs indisputably result in immunosuppression in glioma and facilitate tumor progression [74]. Gielen et al. demonstrated that the MDSC number was elevated in blood samples from GBM patients compared with that in healthy donors [75]. It is also reported that MDSCs are powerful inhibitors of antitumor immune responses via the following mechanisms [74]. MDSCs can suppress the number and function of antigen-presenting cells (APCs, mainly DCs). Additionally, MDSCs can thwart innate and adaptive immunity by inhibiting immune effector cells, including cytotoxic CD8^+^ T cells and NK cells. Also, they can promote the infiltration of other immunosuppressive cells, such as Tregs. Tregs are derived from CD4^+^ T cells lineage and are characterized by the expression of receptors, including typical transcription factor forkhead box P3 (FoxP3) and other receptors that are not particularly specific for Tregs (such as CD25, CTLA-4 and PD-1) [76]. A high frequency of Tregs in the circulating CD4^+^ population is closely associated with T-cell exhaustion, leading to a weak immune response and poor prognostic in the clinic. Hence, natural products targeting MDSCs and Tregs would be beneficial to reversing the glioma immunosuppressive microenvironment.

#### 3.2.1. Sulforaphane

Sulforaphane is a natural compound derived from broccoli sprouts, attractive for its immunomodulatory properties. A study reported that in the glioma-conditioned media in vitro, sulforaphane could decrease the number of MDSCs that protected the glioma cells from immunosurveillance and facilitated tumor progression. The sulforaphane-induced declined number of MDSCs was exerted by inhibiting the transformation from normal monocytes to MDSCs, and simultaneously promoting MDSC differentiation to a mature DC phenotype which promoted T-cell proliferation [77,78]. Furthermore, the inhibition of macrophage migration inhibitory factor (MIF) derived by glioma cells was one of the mechanisms of sulforaphane’s ability to suppress MDSCs. Most importantly, this natural product displayed direct antiglioma activity through oral administration without toxicity to normal monocytes and lymphocytes [77].

#### 3.2.2. Gamabufotalin

Gamabufotalin is one of the active bufadienolide compounds extracted from a traditional Chinese Medicine cinobufacini, which is derived from dried toad venom (Chan Su) from the skin glands of Bufo gargarizans or Bufo melanostictus. Cinobufacini has been investigated to treat various kinds of cancers (such as hepatoma and lung cancer) and exert immunomodulatory effects [79,80,81]. A study showed that gamabufotalin, acting as a key active compound of cinobufacini, possessed a selective cytocidal effect against GBM cells instead of normal cells [81]. In vitro experiments demonstrated that gamabufotalin at an almost non-toxic concentration could efficiently decrease the percentage of CD4^+^CD25^+^Foxp3^+^ Tregs in mitogen-activated peripheral blood mononuclear cells (PBMCs) without impacting the number of CD4^+^ T cells. Thus, gamabufotalin can be used as a promising adjuvant therapeutic agent by inhibiting Tregs to reverse the immunosuppressive microenvironment for glioma immunotherapy [81].

### 3.3. Natural Products Reactivating T Cells and NK Cells

In glioma immunotherapy, researchers have focused on the attempts to reactivate the cytotoxic activity of CD8^+^ T cells and NK cells, and rescue the exhaustion of these effector cells. Many natural products have been found to promote the activation of CD8^+^ T cells and NK cells, thus potentiating immune response and immunotherapeutic efficacy. In addition, natural products can suppress the expression of immune inhibitory checkpoints on T cells to reverse the resistance of glioma immunotherapy. Furthermore, they also can promote the frequency and function of CD4^+^ T cells, which are significant for T cell-mediated adaptive immunity.

#### 3.3.1. Triptolide

Triptolide is an epoxy diterpene lactone compound extracted from traditional Chinese herbs *Tripterygium wilfordii* Hook F. It is reported to inhibit the proliferation and invasion of glioma cells, and enhance temozolomide-induced apoptosis synergistically [82,83]. Zhang et al. cocultured T cells with glioma cells treated with IFN-γ and triptolide to explore the effects of this natural compound on T-cell inhibition in glioma [83]. Amongst, IFN-γ was used to induce the expression of MHC II and PD-L1 on the surface of glioma cells. The results showed that triptolide can reverse the inhibitory effect on CD4^+^ T cells in the glioma immunosuppressive microenvironment, which was consistent with the Flies group’s investigation that the inhibition of CD4^+^ T cells mainly accounts for the tumor immunosuppressive microenvironment [84]. It also downregulated the expression of PD-L1 induced by INF-γ. These results indicated triptolide can be developed as an alternative drug for glioma immunotherapy. Furthermore, triptolide promoted IL-2 secretion and reversed IL-10 inhibition caused by glioma cells.

#### 3.3.2. Ganoderma Lucidum Polysaccharides

Ganoderma lucidum polysaccharides are prospective components in *Ganoderma lucidum* (Curtis) P. Karst., showing various biological activities, including antitumor, antiangiogenesis, antioxidant, anti-inflammatory and immunomodulatory effects [85,86,87,88]. Wang et al. found that Ganoderma lucidum polysaccharides could significantly increase T-cell proliferation and infiltration in tumors and enhance the cytotoxicity of splenic NK cells in a dose-dependent manner in glioma-bearing rats [89]. They also could increase the serum concentration of IL-2, TNF-α and IFN-γ, which contributed to activating immune responses and eliciting antiglioma effects. These results indicated that Ganoderma lucidum polysaccharides could be applied to glioma immunotherapy by regulating the body’s immune response.

### 3.4. Natural Products Modulating Immune-Related Signaling Pathway in Glioma Cells

The action mode of multicomponent-multitarget-multipathway renders natural products better antitumor effects [90]. Apart from the aforementioned effects on immunosuppressive cells and immune effector cells, natural products can also modulate immune-related signaling pathways in glioma cells. In the tumor microenvironment, glioma cells will produce various factors which contribute to the aggressive growth of tumors. Amongst, TGF-β is a common growth factor mainly secreted by glioma cells and surrounding-microglial, which can promote the malignant phenotype of glioblastoma by enhancing proliferation, invasion, angiogenesis and immune-suppressive effects of tumor cells [91]. Accordingly, TGF-β-related signaling pathways remain a potent target for glioma immunotherapy via the regulation of natural products.

#### 3.4.1. Paeoniflorin

Paeoniflorin is a monoterpene glycoside compound derived from traditional Chinese herbs *Paeonia lactiflora* Pall. Previous studies showed that it displays various pharmacological activities, among which the antitumor effects have been studied by increasing research [92,93,94,95,96]. Especially, due to immunoregulation effects and the ability to penetrate the blood-brain barrier quickly, paeoniflorin was investigated for glioma immunotherapy in a preclinical study [97]. Wang et al. proved that paeoniflorin downregulated TGF-β expression and the markers (such as snail, vimentin and N-cadherin) of epithelial-to-mesenchymal transition (EMT) in U87, U251, T98G cell lines [97]. The results indicated that paeoniflorin is a prospective natural therapeutic that can suppress the migration and invasion of glioma cells by inhibiting TGF-β-induced epithelial-to-mesenchymal transition that was crucial to the tumor progression.

#### 3.4.2. Diosmetin

Diosmetin is a flavonoid extracted from traditional Kazakh medicine *Dracocephalum peregrinum* L. and has been previously studied with mainly anticancer, anti-inflammatory, and antioxidative effects [98,99,100,101]. Furthermore, it has been demonstrated to be safe and can induce the apoptosis of cancer cells without damage to normal cells. Wu et al. showed that diosmetin functioned as a suppressor of glioma growth, proliferation and migration in vivo and in vitro, probably for its inhibition of the TGF-β signaling pathway in glioma cells [102]. Low expression of E-cadherin indicated the occurrence of EMT, which led to the increased risk of tumor metastasis and invasion. Diosmetin was demonstrated to facilitate the expression of E-cadherin through the TGF-β pathway, thus suppressing the development of malignant glioma. It indicated that diosmetin might be a candidate for glioma treatment by thwarting the TGF-β signaling pathway.

**Table 1:** Natural products with antitumoral immune modulatory activities for glioma immunotherapy ^1^.

Category of Action Mode	Natural Product	Structural Formula		Origin/ Occurrence	Immune Modulatory Effects on Glioma	Molecular Mechanism	Ref.
Natural products remodeling TAMs	Chlorogenic acid	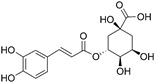		Phenylpropanoid isolated from honeysuckle, eucommia, hawthorn	↑ iNOS, MHC II and CD11c ↓ Arginase and CD206	↑ STAT1 activation ↓ STAT6 activation	[57]
					↑ TAM polarization to the M1 phenotype	/	[59]
					↑ MHC II	/	[55]
	Curcumin	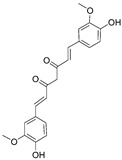		Flavonoid isolated from *Curcuma longa* L.	↑ M1 phenotype (Arginase1^low^, iNOS^high^) ↓ M2 phenotype (Arginase1^high^, iNOS^low^)	↓ Nuclear localization of the NF-κB p50-homodimer that promoted TAMs to M2-type ↑ Activation of the p50/p65 NF-κB	[63]
					↑ TAM polarization to M1 phenotype ↑ Recruitment of NK cells to tumor sites	/	[64]
	Apigenin	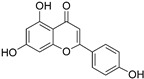		Flavonoid isolated from *Croton betulaster* Müll.	↓ CD206 on microglia ↑ OX-42 and iNOS on microglia	↑ TNF ↓ IL-10	[66]
	Ginsenoside Rg3	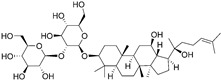		Triterpenoid isolated from *Panax ginseng* C. A. Mey.	↓ M2-type population (CD206^+^) ↑ M1-type population (iNOS^+^)	/	[69]
	Rutin and quercetin	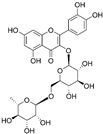	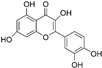	Two flavonoids isolated from various vegetables, fruits, and other plants	↑ Microglial chemotaxis to tumor	↓ mRNA expression for IL-10 and ↑ TNF, CCL2, CCL5, CXCL1, HBGF, IGF and GDNF.	[72]
		Rutin	Quercetin		↑ Microglia polarization toward an inflammatory profile	↑ mRNA expression for IL-1β, IL-6 and IL-18 and ↓ arginase, TGF-β, NOS2 and PTGS2	
Natural products inhibiting MDSCs and Tregs	Sulforaphane	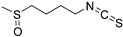		Sulfocompound isolated from broccoli	↓ Transformation from normal monocytes to MDSCs ↑ MDSC differentiation to a mature DC phenotype	↓ Macrophage Migration Inhibitory Factor (MIF) derived by glioma cells	[76]
	Gamabufotalin	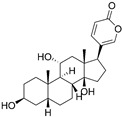		Sterioid isolated from cinobufacini	↓ Percentage of CD4^+^CD25^+^Foxp3^+^ Tregs in mitogen-activated peripheral blood mononuclear cells (PBMCs)	/	[80]
Natural products reactivating T cells and NK cells	Triptolide	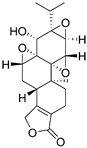		Terpeonoid isolated from *Tripterygium wilfordii* Hook. f.	↓ Inhibitory effect on CD4^+^ T cells in the glioma immunosuppressive microenvironment	/	[82]
	Ganoderma lucidum polysaccharide			Saccharide isolated from *Ganoderma lucidum* (Curtis) P. Karst.	↑ T-cell proliferation and infiltration in tumors ↑ Cytotoxicity of splenic NK cells	/	[88]
Natural products modulating immune-related signaling pathway in glioma cells	Paeoniflorin	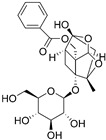		Monoterpene glycoside isolated from *Paeonia lactiflora* Pall.	↓ TGF-β-induced Epithelial-to-mesenchymal transition	/	[96]
	Diosmetin	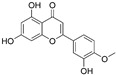		Flavonoid isolated from *Dracocephalum peregrinum* L.	↓ TGF-β signaling pathway in glioma cells ↑ E-cadherin	/	[101]

^1^ Arrows indicate an increase (↑) or a decrease (↓).

As mentioned above, natural product-mediated glioma immunotherapy exhibits antitumor effects by reversing one or more pathways of the tumor immunosuppressive microenvironment. It mainly includes remodeling immunosuppressive cells (such as TAMs, MDSCs and Tregs), reactivating immune effector cells (T cells and NK cells), and modulating TGF-β-related signaling pathways in glioma cells. The above studies demonstrated a broad prospect for glioma immunotherapy of natural products in the future.

## 4. Conclusions and Future Perspectives

The effects of natural products on the immune system are varied and complex, and the effects of different natural products on various immune cells are different. Natural products contain a rich variety of chemical components, including alkaloids, polysaccharides, glycosides and flavonoids, etc. These chemicals have a variety of biological functions and a wide range of effects on the immune system. TCM can reshape the tumor microenvironment to regulate the immune system, for example, can reduce the number of M2 type TAMs and Treg cells, and macrophages increased the activation of T cells. In China, traditional Chinese medicine has a long history of clinical applications and the promotion of antiglioma immunity by some traditional Chinese medicines will provide a promising method for the treatment of glioma.

Although natural products may play a significant role in the immunotherapy of glioma under their unique advantages, they are still far from reaching the ideal therapeutic effect. Due to the existence of the blood-brain barrier, it is difficult for natural products to enter the glioma site and achieve a certain accumulation. Based on this, natural products mainly regulate the body and the entire immune system, but the direct killing effect on glioma is limited. Therefore, how to reverse the immunosuppressive microenvironment in the brain with natural products to enhance the effect of immunotherapy will become key. In fact, the nano-drug delivery system has already played an important role in the treatment of glioma. The nano-drug delivery system can help natural products cross the blood-brain barrier and be released at the glioma site, so as to achieve the targeting of the glioma site and realize the direct contact of natural products with glioma cells. And with a slow-release effect, it can improve the bioavailability of natural products. Chemotherapy is still an important method in the clinical treatment of glioma, however, chemotherapy combined with immunotherapy and adjuvant therapy with natural products may be a potential treatment option for glioma. Combined with the advantages of natural products, glioma can be better handled by reshaping the microenvironment of glioma and regulating the immunosuppressive microenvironment.

## Data Availability

Not applicable.
